# Genetic variations *in chromodomain helicase DNA-binding protein 5*, gene-environment interactions and risk of sporadic Alzheimer’s disease in Chinese population

**DOI:** 10.18632/oncotarget.23791

**Published:** 2018-05-18

**Authors:** Xiao Zhu, Haibing Yu, Qin Xiao, Jianhao Ke, Hongmei Li, Zhihong Chen, Hongrong Ding, Shuilong Leng, Yongmei Huang, Jingting Zhan, Jinli Lei, Wenguo Fan, Hui Luo

**Affiliations:** ^1^ Key Laboratory of Medical Molecular Diagnosis, Dongguan Scientific Research Center, Guangdong Medical University, Dongguan, China; ^2^ Department of Blood Transfusion, Peking University Shenzhen Hospital, Shenzhen, China; ^3^ Tropical Crops Department, Guangdong AIB Polytechnic, Guangzhou, China; ^4^ Department of Human Anatomy, Guangzhou Medical University, Guangzhou, China; ^5^ Department of Anatomy and Physiology, Guanghua School of Stomatology, Sun Yat-sen University, Guangzhou, China; ^6^ Institute of Marine Medicine Research, Guangdong Medical University, Zhanjiang, China; ^7^ Institute of Bioinformatics, University of Georgia, Athens, GA, USA

**Keywords:** Alzheimer's disease, case-control studies, CHD5, gene-environment interactions, single-nucleotide polymorphisms, Gerotarget

## Abstract

CHD5 is an essential factor for neuronal differentiation and neurodegenerative diseases. Here, the targeted next generation sequencing and TaqMan genotyping technologies were carried out for *CHD5* gene in a two-staged case-control study in Chinese population. The genetic statistics and gene-environment interactions were analyzed to find certain risk factors of Alzheimer's disease. We found intronic rs11121295 was associated with the risk of Alzheimer's disease at both stages including combined cohorts. This risk effect presented consistently significant associations with the alcoholic subgroups at both all stages in the stratified analysis. The gene-environment interactions further supported the above findings. Our study highlighted the potential role of *CHD5* variants in conferring susceptibility to sporadic Alzheimer's disease, especially modified its risk by alcoholic intake.

## INTRODUCTION

Alzheimer's disease, a progressive neurodegenerative disorder, is one of the major dementia in elderly people that usually starts slowly and worsens over time [1]. It causes a tremendous societal challenge because the popularity of Alzheimer's disease continues to rise. It affects about 6% of people over 65 years of age, and there are approximately 30 million people worldwide with Alzheimer's disease [2].

The cause of Alzheimer's disease is poorly understood. About 70% of the risk is believed to be genetic with many genes usually involved [3]. Recent studies have proved that a number of polymorphisms impact on the progression of patients with Alzheimer's disease [4, 5]. However, more than 90% of cases are scattered, as a result, only a few percentage points have clear genetic causes. One of the most frequent genetic modifications depicted is the deletion of the short arm of chromosome 1 found in around 35% of neuroblastoma [6]. Other risk factors include a history of head injuries, depression and anxiety, or hypertension.

*Chromodomain helicase DNA-binding protein 5 (CHD5)* located in the human chromosome 1p36.31. CHD5 belongs to the ATP-dependent chromatin remodeling protein snf2 DNA methylase/helicase (SNF2) superfamily which is one of the nine members of the chromodomain helicase DNA-binding (CHD) family of enzymes [7, 8]. According to the latest report, the expression of CHD5 was in several brain regions and neurons [9, 10]. And then CHD5 directly regulated the targets including genes which are important for aging, Alzheimer's disease, and neuronal development. Also, intellectual impairment has been coupled with the deletion of a region of chromosome 1 near *CHD5* [11]. However, it remains to be confirmed that the specific role of CHD5 in brain development and function. Based on all of these findings, we set out to investigate whether *CHD5* single-nucleotide polymorphisms (SNPs) were associated with risk of Alzheimer's disease in a two-stage case-control study from China.

## RESULTS

**Figure 1:**
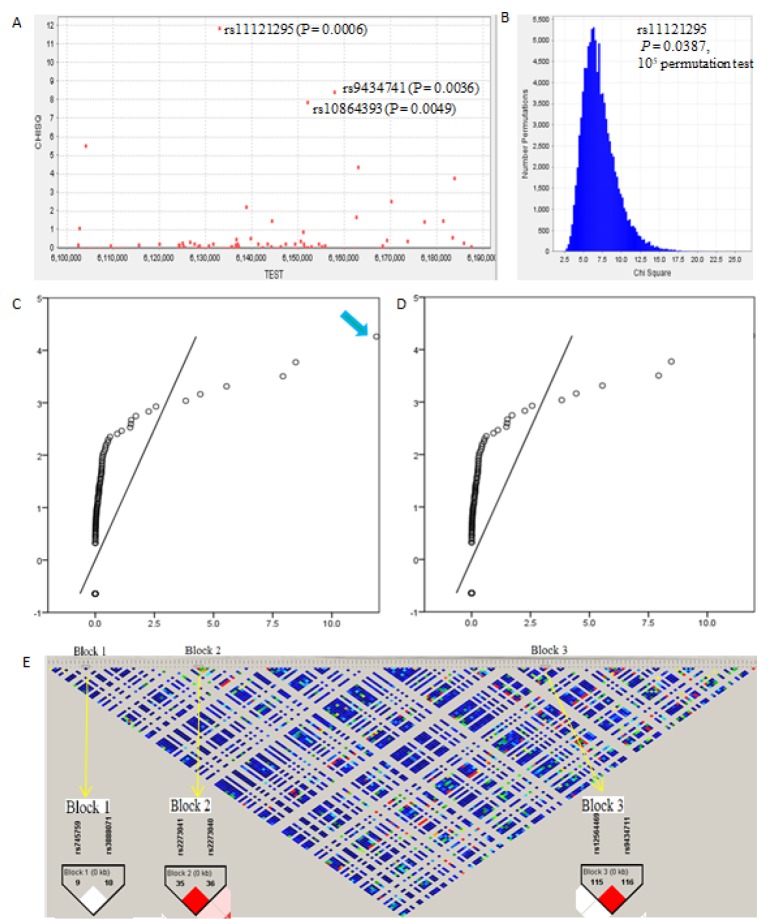
The association analysis of CHD5 variants with Alzheimer's disease risk and genetic mapping in the discovery study **A.** Manhattan plot. The *Chi* square values were for the association of each variant with Alzheimer's disease risk, from two-sided Cochran-Armitage tests for trend. **B.** 10^5^ permutation tests for the association analysis carried out from haploview 4.0 program (*Bioinformatics 2005;21:263-5*).**C.** and **D.** Q-Q plots for the test statistics of observed Chi-square values against expected Chi-square values (D, removing rs11121295). **E.** Linkage disequilibrium mapping. Block 1 includes SNP9~SNP10 (rs745759 and rs3888071). Block 2 includes SNP35~SNP36 (rs2273041 and rs2273040). Block 3 includes SNP115 and SNP116 (rs12564469 and rs9434711).

In the discovery study, the genotype distributions of 164 candidate variants in the case and control groups are shown in Supplementary Table 1. We found three variants perhaps were associated with Alzheimer's disease risk (rs11121295, *P*_for alleles_ = 6.00×10^-4^; rs10864393, *P*_for alleles_ = 0.0049; and rs9434741, *P*_for alleles_ = 0.0036; Figure 1A). Even after 10^5^ permutation tests, there was still remain significance for rs11121295 (*P* = 0.0387; Figure 1B). The observed *X*^2^ values with the distribution of null hypothesis were deviated from the expectations at higher value of approximately 4.3 (Figure 1C). But it lost its significance after removing rs11121295 (Figure 1D).

Indeed, rs11121295 GG genotype and rs10864393 AA genotype all showed significance of risk with Alzheimer's disease in the discovery cohort (RR_Hom_ = 2.67, *P*_Hom_ = 3.460×10^-4^ for rs11121295; RR_Hom_ = 0.44, *P*_Hom_ = 0.003 for rs10864393; Table 1). These risk qualities increased in a dose-dependent manner as genotypes increased or decreased (*P*_trend_ = 5.495×10^-4^ for rs11121295, and .004 for rs10864393).

**Table 1:** Association results of the selected three SNPs from next generation sequencing in discovery, replication and combined studies

SNPs	Cases DD/DA/AA	Controls DD/DA/AA	RR_Het_ (95% CI)	*P*_Het_	RR_Hom_ (95% CI)	*P*_Hom_	*P*_trend_
Discovery							
rs11121295	51/99/55	89/130/36	1.33 (0.86-2.05)	.197	2.67 (1.55-4.59)	3.460×10^-4^	5.495×10^-4^
rs10864393	55/114/36	49/133/73	0.76 (0.48-1.21)	.249	0.44 (0.25-0.77)	.003	.004
rs9434741	8/60/137	12/112/131	0.80 (0.31-2.07)	.651	1.57 (0.62-3.96)	.337	.003
Replication							
rs11121295	105/198/94	174/262/74	1.25 (0.92-1.70)	.147	2.11 (1.43-3.11)	1.603×10^-4^	2.760×10^-4^
rs10864393	76/225/96	94/262/154	1.06 (0.75-1.51)	.736	0.77 (0.52-1.15)	.197	.137
rs9434741	20/173/204	24/236/250	0.88 (0.47-1.64)	.687	0.98 (0.53-1.82)	.947	.605
Combined							
rs11121295	156/297/149	263/392/110	1.28 (1.00-1.64)	.054	2.28 (1.67-3.13)	2.445×10^-7^	6.463×10^-7^
rs10864393	131/339/132	143/395/227	0.94 (0.71-1.24)	.645	0.64 (0.46-0.87)	.005	.003
rs9434741	28/233/341	36/348/381	0.86 (0.51-1.45)	.573	1.15 (0.69-1.93)	.593	.031

D,derived alleles; A, ancestral alleles; Het, heterozygous variants; Hom, homozygous variants.

P_trend_ were calculated in logistical regression models with adjustment for age, gender, smoking and drinking status.

We identified three blocks with high linkage disequilibrium (Figure 1E). Block 1 includes SNP9~SNP10 (rs745759 and rs3888071). Block 2 includes SNP35~SNP36 (rs2273041 and rs2273040). Block 3 includes SNP115 and SNP116 (rs12564469 and rs9434711). The results of the haplotype-based case-control study between the Alzheimer's disease and control groups are shown in Supplementary Table 2. We found that a haplotype AG in block 3 showed a significant association with Alzheimer's disease risk (*P* = 1.620×10^-5^). Nevertheless, it is actually difficult to determine Alzheimer's disease risk, because the proportions of the frequencies of haplotype AG were all too small (0.055 in cases and 0.005 in controls).

According to the discovery study, SNPs rs11121295, rs10864393 and rs9434741 were selected for the replication and combined studies. Genotype frequencies distribution of rs11121295 was significantly different between the two groups not only in replication study (RR_Hom_ = 2.11, *P*_Hom_ = 1.603×10^-4^ and *P*_trend_ = 2.760×10^-4^) but combined study (RR_Hom_ = 2.28, *P*_Hom_ = 2.445×10^-7^ and *P*_trend_ = 6.463×10^-7^; Table 1).

This association of rs11121295 with the Alzheimer's disease risk was further evaluated by subgroups of age, sex, education, smoking and drinking status with replication and combined studies (Table 2). We found that those carried rs11121295 had a significantly increased risk, and this risk was more obvious not only in the older group (discovery: *P* = 0.005, and *P*_i (P2/P1)_ = 0.011; replication: *P* = 8.700×10^-4^, and *P*_i (P2/P1)_ = 0.003), but in the drinking group (discovery: *P* = 6.941×10^-4^, and *P*_i__(P2/P1)_ = 0.008; replication: *P* = 0.002, and *P*_i (P2/P1)_ = 0.049) in all two case-control studies, especially their combined study (*P* = 1.508×10^-5^, and *P*_i (P2/P1)_ = 8.621×10^-5^ for older group; and *P* = 7.164×10^-6^, and *P*_i__(P2/P1)_ = 8.755×10^-4^ for drinking group).

**Table 2:** Stratification analysis for associations between rs11121295 and AD risk in the discovery, replication and combined studies

Variables	Discovery study				Replication study				Combined study			
	Cases/controls		OR (95% CI)	*P*	Cases/controls		OR (95% CI)	*P*	Cases/controls		OR (95% CI)	*P*
	GG 55/36	AA+AG 150/219			GG 94/74	AA+AG 303/436			GG 149/110	AA+AG 453/655		
Age (ys)												
≤60	Feb-33	20/183	0.56(0.12-2.49)	0.435	Apr-68	39/366	0.55(0.19-1.60)	0.266	6/101	59/549	0.55(0.23-1.31)	0.174
>60	53/3	130/36	4.89(1.44-16.58)	0.005	90/6	264/70	3.98(1.67-9.47)	8.700×10-4	143/9	394/106	4.28(2.11-8.67)	1.508×10-5
*P_i_*				0.011				0.003				8.621×10-5
Gender												
Females	29/11	85/80	2.48(1.16-5.30)	0.017	47/18	161/149	2.42(1.34-4.35)	0.003	76/29	246/229	2.44(1.53-3.88)	1.220×10-4
Males	26/25	65/139	2.22(1.19-4.15)	0.011	47/56	142/287	1.70(1.10-2.63)	0.017	73/81	207/426	1.86(1.30-2.65)	6.325×10-4
*P*_i_				0.647				0.176				0.193
Education												
Less	34/18	108/116	2.03(1.08-3.80)	0.026	42/26	145/149	1.66(0.97-2.85)	0.064	76/44	253/265	1.81(1.20-2.72)	4.207×10-3
More	21/18	47/87	2.16(1.05-4.45)	0.035	52/43	140/229	1.98(1.25-3.12)	0.003	73/61	187/316	2.02(1.38-2.97)	2943×10-4
*P*_i_				0.743				0.047				0.07
Smoking												
Never	33/27	115/165	1.97(1.13-3.41)	0.015	59/77	208/262	0.97(0.66-1.42)	0.857	96/104	323/427	1.22(0.89-1.67)	0.212
Ever+current	18/9	35/47	2.69(1.08-6.69)	0.031	25/29	92/116	1.09(0.60-1.98)	0.786	43/38	127/163	1.45(0.89-2.38)	0.138
*P_i_*				0.484				0.917				0.651
Drinking												
Never	29/26	113/168	1.66(0.93-2.96)	0.086	54/55	189/297	1.54(1.02-2.34)	0.041	83/81	302/465	1.58(1.13-2.21)	0.008
Ever+current	26/7	37/47	4.72(1.85-12.07)	6.941×10-4	41/18	98/111	2.58(1.39-4.78)	0.002	67/25	135/158	3.14(1.88-5.24)	7.164×10-6
*P*_i_				0.008				0.049				8.755×10-4

*P, P* value for haplotype model, which obtained in logistic regression with adjustment for age, sex, smoking status and drinking status.

*P_i_*, means *P_2_/P_1_* or *P_1_/P_2_*.

For Alzheimer's disease risk in older group, the false-positive report probability (FPRP) values of rs11121295 GG were below 0.20 for the assigned prior probability (0.008 for the prior probability of 0.1 in the discovery study; 0.005, 0.010 for the prior probabilities of 0.1, 0.01, respectively in the replication study; and 0.002, 0.009, 0.081 for the prior probabilities of 0.1, 0.01, 0.001 respectively in the combined study). For Alzheimer's disease risk in drinking group, when the assumption of prior probability was 0.1, significant findings were noteworthy in the replication study but in the combined study (prior probability 0.011 and 0.004, respectively). Moreover, when the assumption of prior probability was 0.01, this prominent association was just found in the combined study (prior probability 0.010; Table 3).

**Table 3:** FPRP values for associations between AD risk and rs11121295 frequencies (GG vs. AA+AG)

Variables	Statistical	Prior probability			
	power^a^	.1	.01	.001	.0001
AD risk in >60 years old group					
Discovery study	.327	.008	.029	.443	.742
Replication study	.552	.005	.010	.288	.539
Combined study	.815	.002	.009	.081	.205
AD risk in drinking group					
Discovery study	.434	.254	.418	.585	.737
Replication study	.276	.011	.366	.649	.813
Combined study	.691	.004	.010	.203	.516

Lastly, gene-environmental interactions were studied. We found that the age was the best one-factor model with the highest cross-validation consistency (CVC) (94/100, 93/100, 97/100) and the lowest prediction error (0.392, 0.383, 0.336) in three statistical analyses respectively (Table 4). The age plus rs11121295 was the best model for two-factors, with the highest CVC (93/100, 96/100, 97/100) and the lowest prediction error (0.323, 0.372, 0.312) in three groups respectively. Interestingly, the model with 6-factors had a maximum CVC (100/100, 99/100, 100/100) and a minimum prediction error (0.261, 0.211, 0.134) in three groups respectively, which presents a model with better prediction than one factor.

**Table 4:** MDR analysis for the prediction of AD risk with and without rs11121295

Best interaction models	Cross-validation	Average prediction error	*P^a^*
Distcovery study			
1	94/100	0.392	<.0001
1,2	93/100	0.323	<.0001
1,2,3	97/100	0.354	<.0001
1,2,3,4	98/100	0.225	<.0001
1,2,3,4,5	90/100	0.329	<.0001
1,2,3,4,5,6^b^	100/100	0.261	<.0001
Replication study			
1	93/100	0.383	<.0001
1,2	96/100	0.372	<.0001
1,2,3	92/100	0.394	<.0001
1,2,3,4	97/100	0.373	<.0001
1,2,3,4,5	89/100	0.256	<.0001
1,2,3,4,5,6^b^	99/100	0.211	<.0001
Combined study			
1	97/100	0.336	<.0001
1,2	97/100	0.312	<.0001
1,2,3	90/100	0.375	<.0001
1,2,3,4	96/100	0.213	<.0001
1,2,3,4,5	97/100	0.168	<.0001
1,2,3,4,5,6^b^	100/100	0.134	<.0001

Labels: 1, age; 2, rs11121295; 3, drinking status; 4, education; 5, gender; 6, smoking status.

a, *P* value for 1000-fold permutation test. b, the best model with maximum cross-validation consistency and minimum prediction error rate.

## DISCUSSION

The genome-wide association studies have found some chromosome regions including rare variants that appear to affect Alzheimer's disease risk [3, 12, 13]. The potential genes including CD2AP, SlC24A4, HLA-DRB5, etc. For example, variants in the *TREM2* gene might be associated with a three to five times higher risk [14]. In our study, we reported the positive association between the rs11121295 homozygous variant and Alzheimer's disease not only in the discovery cohort but in the replication and combined studies with targeted next generation sequencing and TaqMan genotyping technologies, which has not been reported before.

The quantile-quantile (Q-Q) plots are plots of two quantiles against each other, which could examine if two data sets have roots in the same distribution. If the two sets of data have a common distribution, the points will fall on that reference line of forty-five degree angle. In our study, a significant change appeared after taking away rs11121295, further indicating that it is the logical risk locus.

The 1p36 is frequently deleted in neural crest tumors including neuroblastomas, raising that this region may contribute to the neurologic and developmental issues. *CHD5* gene contains 42 exons and spans more than 78 kb, it *acts on acid anhydrides in phosphorus-containing anhydrides annotated by g*ene ontology. A downstream gene CDKN2A could regulate the P53 pathway in particular, which in turn, impedes cell proliferation [9]. These suggested CHD5 plays an important role in the neurogenesis and development by activating expression of specific genes promoting neuron terminal differentiation.

Moreover, this study first explored the potential gene-environment interactions by stratification (addressed ORs) adding high-order interactions assessed by FPRP and multiple dimension reduction (MDR) analyses with five known risk factors (age, gender, education, smoking and drinking status), which suggested that older age ( > 60 years), drinking, as well as rs11121295, contributed to an increased Alzheimer's disease risk at different levels. These experiment-wise results further revealed that potential gene-environment interactions seem to predispose to Alzheimer's disease. Although the FPRP can yield serious inferential errors, the FPRP was still proposed as a Bayesian prophylactic against false reports of significant associations [15] .

In developed countries, dementia is one of the most financially costly diseases. It led to about 1.9 million deaths every year. Of them, the cause of 60% to 70% of cases is Alzheimer's disease. Its most common symptoms include language barrier, orientation disorder, mood swings, loss of self-control and behavioural issues. Alzheimer's disease most often begins in people over 60 years and older, although about 4% of cases are early-onset which begin before this. Growing evidence from previous epidemiological studies and meta-analysis has firmly established the important role of older age as a causal factor for Alzheimer's disease [16–18]. A report from the World Health Organization deemed that prevalence rates in developing regions like China are lower than that in developed regions [19]. Chan *et al.* reported Alzheimer prevalence was estimated to be 2.6% in the 65-69 age group, and 60.5% in the age 95-99 years in China in 2010 [20]. This suggested the incidence of Alzheimer's disease increases with age. Though unmatched ages with cases were used in this case-control study would lead to incorrect risk assessment, a proper statistical analysis can well control for differences between the groups. Estimates of effects can be statistically adjusted for covariates that may be different between cases and controls. Logistic regression is mostly used, the weighted one being known as the one that controls for confounders for example. This may still be good enough for the purpose, therefore, the results are still quite valuable.

The genetic heritability of Alzheimer's disease ranges from 49% to 79% based on family studies, and about 0.1 percent of the cases are autosomal dominant inheritance, which have an onset before age 65 [21–23]. But most cases do not exhibit autosomal-dominant inheritance and are termed sporadic Alzheimer's disease, in which genetic and environmental differences may act as risk factors. In our study, drinking status with *CHD5* rs11121295 variant presented a promising association with Alzheimer's disease risk compared with non-drinking carrying that genotype, which indicated that this variant might act in response to drinking, and true associations might be detected by alcoholic stimulation. It has been shown that alcohol could modulate the effect of various cytokines, receptors or neuroimmune signaling in brain, such as midkine (MDK) [24], PPARgamma receptor [25], and HMGB1, miRNA and TLR receptors [26]. Therefore, we speculated, alcohol intake might trigger proinflammatory events through their induction of oxidative stress and extensive inflammation.

The process of Alzheimer's disease is related with tangles and plaques in the brain [2, 27]. The standard diagnosis is in view of the medical history, cognitive examinations, imaging check and blood testing to preclude other possible causes. However, evidence to support the proposals is usually not sufficient. Therefore, clinical genetic examinations may play an important role in the future.

In summary, we identified rs11121295 variant in *CHD5* gene that was highly associated with risk of developing Alzheimer's disease from Chinese descent. CHD5 is a nuclear protein which forms a nucleosome remodeling and deacetylation (NuRD) complex, and it may affect the brain neurons in the level of chromatin remodeling and gene transcription [28]. Nevertheless, we do not yet understand the mechanisms of how it is impacting on the expression of CHD5 protein in the brain. Hence, it will be much more interesting as further investigations of this gene are implemented, not only in Alzheimer's disease patients but also in healthy commons.

## MATERIALS AND METHODS

### Subjects

In the first step, 205 unrelated Alzheimer's disease patients and 255 healthy control people who had no history of Alzheimer's disease and other conspicuous diseases from Zibo Center Hospital in North China were included as the discovery study. Then, a replicative study including 397 Alzheimer's disease cases and 510 controls (from Guangdong Provincial People's Hospital, Guangzhou Brain Hospital and Peking University Shenzhen Hospital in South China) was carried out. Finally, the above both were included as the combined study. At recruitment, each study participant (or his/her relative) was interviewed *via* a structured questionnaire, to obtain information on demographic characteristics, habits of cigarette smoking and alcohol drinking, as well as personal and family history of major chronic illnesses. A pack of cigarettes was defined as 20 cigarettes in China. “Ever or current smoking” were defined by valuing subjects who had smoked more than 5 packs in their whole life before the date of diagnosis for cases, or before the date of the interview for controls [29]. “Ever or current drinking” as having consumed alcoholic beverages ≥1 time/week for ≥ 6 months previously; otherwise, they were defined as non-drinkers [30]. One drink was regarded as 30 g of spirits (12.9 g of ethanol), 103 g of wine (12.3 g of ethanol), or 360 g of beer (12.6 g of ethanol) [30]. Less education implied that he only accept the primary school education or less [31]. The main features of the subjects included are summarized in Table 5. The Ethics committee of Guangdong Medical University authorized the protocol of this study. The study also adhered to tenets in the declaration of Helsinki.

**Table 5:** Demographics of patients with Alzheimer’s disease and controls in three study cohorts

Characteristics	Discovery cohort			Replicative cohort			Combined cohort		
	Cases	Controls	*P*	Cases	Controls	*P*	Cases	Controls	*P*
No.of subjects	205	255		397	510		602	765	
Origin	NC	NC		SC	SC		NC+SC	NC+SC	
Females, n (%)	114 (55.61)	91 (35.69)	<.001^a^	208 (52.39)	167 (32.75)	<.001^a^	322 (53.49)	258 (33.73)	<.001^a^
Age (mean ± SD)	73.5±10.2	41.5±9.1	<.001^b^	73.0±10.2	47.2±10.7	<.001^b^	73.2±10.3	44.8±10.3	<.001^b^
Less education, n (%)	137 (66.83)	134 (52.55)	.002^a^	187 (47.10)	175 (34.31)	.003^a^	324 (53.82)	309 (40.39)	<.001^a^
missing	0	16		18	63		18	79	
Cigarette smoking, n (%)	53 (25.85)	56 (21.96)	.417^a^	117 (29.47)	145 (28.43)	.871^a^	170 (28.24)	201 (26.27)	.573^a^
missing	0	7		13	26		13	33	
Alcohol drinking, n (%)	63 (30.73)	54 (21.18)	.030^a^	139 (35.01)	129 (25.29)	.003^a^	202 (33.55)	183 (23.92)	<.001^a^
missing	0	7		15	29		15	36	
Hypertention, n (%)	26 (12.68)	0		43 (10.83)	0		69 (11.46)	0	
Stroke, n (%)	4 (1.95)	0		7 (1.76)	0		11 (1.83)	0	
Diabetes, n (%)	30 (14.63)	0		51 (12.85)	0		81 (13.46)	0	

NC, North China; SC, South China; SD, standard deviation.

^a^Pearson Chi-square test. ^b^Mann-Whitney U-test.

### Targeted sequencing, variants selection and genotyping

Genomic DNA was extracted from the whole blood leukocytes. We sequenced whole *CHD5* gene with next generation sequencing technology (Illumina Genome Analyzer) in 255 controls and 205 Alzheimer's disease samples. A targeted resequencing study was performed on the Illumina platform with pair-end 90 bp reads. Following the manufacturer's instructions, shotgun libraries were built from 5 microgram of genomic DNA, and genomic DNA diluted in Tris-EDTA buffer was sheared into about 500-bp fragments. The DNA fragments were subsequently tailed with A. Then the Illumina sequencing adaptors were ligated to the samples. Finally, the adaptor-linked fragments were enriched *via* PCRs. The prepared library was subsequently hybridized to capture probes. The captured fragments were then amplified with the following protocol: incubation at 95 °C for 5 min followed by 25 cycles of 95 °C for 15 s, 56 °C for 30 s and 72 °C for 60 s and a final extension at 72 °C for 8 min. PCR products were purified and finally sequenced with standard 2 × 90-bp paired-end reads on the Illumina HiSeq 2000 sequencer.

The reads were aligned to the reference genome hg19 (NCBI build 37.1) (NCBI build 37.1) [32]. Single-nucleotide variants that met any of the following criteria were then filtered: *P* for Hardy-Weinberg equilibrium < 10^−4^ [33], duplicated pair-end reads, overall depth ≤ 8×, copy number variant ≥ 2, or SNP within 10 bp of a gap. In this evaluation, we only considered the qualified SNPs, thus yielding a 164-SNPs set, which will be used as the primary case-controls study.

We analyzed the associations of variants (allele frequencies > 1%) and Alzheimer's disease risk. Only three variants (rs11121295, rs10864393 and rs9434741) entered the next step study for their lower *P* values. Then, genomic DNAs from all the other subjects (510 controls and 397 cases) were genotyped by TaqMan probes in Applied Biosystems ABI 7500 Fast System (Forster City, CA) for the above selected three variants. The PCR of samples heated to 95°C for 10 min followed by 40 cycles of 92°C for 15s and 60°C for 1 min.

### Statistical analysis

Chi-square test and Mann-Whitney U-test were used to assess the difference of demographics between cases and controls. The genotype distributions in controls were analyzed with Hardy-Weinberg equilibrium (*P*_HWE_ > 0.01).

Haplotype estimation and permuting association analysis were executed with Haploview program [34] for 10^5^ permutations (the ‘Single Markers Only’ option was used) , in which the subjects’ phenotypes were randomly realigned. By convention if *P* < 0.05, the difference was considered statistically significant. The Q-Q plot was then performed to check the distributions of *P* value. We used homozygote (DD *vs*. AA) and heterozygote (DA *vs*. AA), as the models (D-derived alleles and A-ancestal alleles). Logistic regression was used to test the association, which adjusted for gender, age, education, smoking and drinking. A dose-dependent effect was assessed by the trend test of odds ratios (ORs). The variant(s) (which *P* for genotypes < 0.0003_(0.05/164)_ ) will be entering the next replication study. The FPRP and MDR program [35] was used to evaluate the possible high-order gene-environment interaction. The minimum average prediction error and the maximum CVC were required for the best candidate interaction model. SPSS 22.0 for windows (SPSS, Chicago, IL) and R scripts (3.0.2 Suite) were performed in the statistical analyses.

This work was supported by National Natural Science Foundation of China (81541153); Guangdong Provincial Science and Technology Programs (2015A050502048, 2016A050503046, 2014A020212295, and 2014A020212653); Science and Technology Research Project in Dongguan City (2013508152011 and 2013508152002).

## SUPPLEMENTARY MATERIALS FIGURES AND TABLES




